# The Antiarrhythmic Drug, Dronedarone, Demonstrates Cytotoxic Effects in Breast Cancer Independent of Thyroid Hormone Receptor Alpha 1 (THRα1) Antagonism

**DOI:** 10.1038/s41598-018-34348-0

**Published:** 2018-11-08

**Authors:** Mitchell J. Elliott, Katarzyna J. Jerzak, Jessica G. Cockburn, Zhaleh Safikhani, William D. Gwynne, John A. Hassell, Anita Bane, Jennifer Silvester, Kelsie L. Thu, Benjamin Haibe-Kains, Tak W. Mak, David W. Cescon

**Affiliations:** 1 The Princess Margaret Cancer Centre, University Health Network, Toronto, Canada; 2 Sunnybrook Health Science Centre, Odette Cancer Centre, Toronto, Canada; 3 Division of Medical Oncology, Department of Medicine, University of Toronto, Toronto, Canada; 4 McMaster University, Hamilton, Canada

## Abstract

Previous research has suggested that thyroid hormone receptor alpha 1 (THRα1), a hormone responsive splice variant, may play a role in breast cancer progression. Whether THRα1 can be exploited for anti-cancer therapy is unknown. The antiproliferative and antitumor effects of dronedarone, an FDA-approved anti-arrhythmic drug which has been shown to antagonize THRα1, was evaluated in breast cancer cell lines *in vitro* and *in vivo*. The THRα1 splice variant and the entire receptor, THRα, were also independently targeted using siRNA to determine the effect of target knockdown *in vitro*. In our study, dronedarone demonstrates cytotoxic effects *in vitro* and *in vivo* in breast cancer cell lines at doses and concentrations that may be clinically relevant. However, knockdown of either THRα1 or THRα did not cause substantial anti-proliferative or cytotoxic effects *in vitro*, nor did it alter the sensitivity to dronedarone. Thus, we conclude that dronedarone’s cytotoxic effect in breast cancer cell lines are independent of THRα or THRα1 antagonism. Further, the depletion of THRα or THRα1 does not affect cell viability or proliferation. Characterizing the mechanism of dronedarone’s anti-tumor action may facilitate drug repurposing or the development of new anti-cancer agents.

## Introduction

Despite the successful development of several new classes of therapies for breast cancer, this disease remains the second most common cause of cancer related death in women^1^. While much effort has been devoted to studying and targeting of well-recognized breast cancer drivers, including the estrogen receptor (ER), HER2 receptor, and the PI3K/AKT/mTOR pathways, many aspects of breast cancer biology, which could offer new treatment approaches, remain relatively unexplored. Among these is the role of thyroid hormones and thyroid hormone receptors. The thyroid hormones, thyroxine (T4) and triiodothyronine (T3), are iodine-based hormones produced in the thyroid gland. They serve as important endocrine hormones which regulate multiple cellular processes including metabolism and proliferation^2–5^. Biological effects are regulated by the two classes of thyroid hormone receptors (THRs), alpha (α) and beta (β)^6^, which are homologous ligand-dependent transcription factors that regulate distinct cellular pathways^7–11^.

Previously, work has demonstrated that thyroid hormone signaling is involved in tumor suppression as well as carcinogenesis^12,13^. Thyroid hormone receptors have been shown to antagonize ras-induced proliferation and block fibroblast transformation by both ras and v-src^14^. However, T3 acting through THR signaling has also been shown to increase proliferation and enhance estrogen-mediated growth in immortalized breast cancer cell lines^12^. The specific role of each thyroid receptor subtype in various cancers has yet to be elucidated^15,16^, likely due to the complexity of both genomic and non-genomic actions of THRs^2,17^, differential expression in different human tissues^18^, as well as the potential for cross-talk with estrogen signaling pathways^19–21^. This may be further confounded by circulating levels of thyroid hormones^22^.

There are three recognized functional isoforms of the THRα receptor, THRα1, THRα2, and THRα3 while there are four mRNA isoforms^7^. Recently, a retrospective cohort study in breast cancer patients demonstrated that high tumor THRα1 expression was associated with shorter 5-year survival, particularly when the expression of ‘favorable’ THRα2 was concomitantly low^23^. Other studies have also demonstrated prognostic associations of THRα1 and THRα2 with patient outcomes^24,25^. However, the effect of modulation of THRα and specific isoforms in breast cancer has not been characterized.

It is hypothesized that the prognostic associations of THRs are related to the underlying biology governing the receptors. While THRα1 avidly binds thyroid hormone and mediates its downstream effects, THRα2 lacks a ligand-binding domain and has been described to oppose thyroid-mediated transcription of downstream targets^13^. The effects of THRα3 are not well characterized, but it also lacks a ligand binding domain^7^. While *in vitro* and *in vivo* data confirming this physiology in the setting of breast and other cancers is lacking, it is possible that THRα1 may promote thyroid-mediated breast cancer proliferation and THRα2 may oppose it. These opposing roles might explain previously observed and seemingly paradoxical roles of the THRα pathway in cancer development and progression.

The prognostic data suggests that modulation of the THRα pathway may have therapeutic potential in breast and other cancers^15,23,26^ particularly if specific isoforms can be targeted. In support of this premise, modulation of THRα1 isoform expression in adipose derived stem cells affects expression of genes governing cell cycle and proliferation^27^. Several drugs are known to interact with thyroid hormone receptors in various tissues. Dronedarone, a class III antiarrhythmic drug approved by the Food and Drug Administration (FDA) and Health Canada for the treatment of supraventricular tachyarrhythmia, exhibits preferential antagonism of THRα1 over THRβ1 receptors *in vitro* and *in vivo*^28^. Also, the ability of dronedarone to reduce THRα1 and downstream target expression has been characterized in cardiac myocytes^29^. Similar effects have been observed with amiodarone, a chemically related small molecule^30^. In addition to its clinical availability, the pharmacokinetics of dronedarone have also been well-characterized in animal models, allowing for ease of administration^31,32^.

Based on the known functional roles of these isoforms and the available epidemiologic data in breast cancer patients, the selective antagonism of THRα1 presents a potential opportunity to repurpose dronedarone as an anti-cancer agent. We therefore sought to evaluate the anti-cancer effects of dronedarone and to characterize the potential of targeting THRα1 in human breast cancer models.

## Results

### THRα and THRα1 overexpression is associated with shorter overall survival in breast cancer patients in The Cancer Genome Atlas (TCGA) dataset

**Figure 1:**
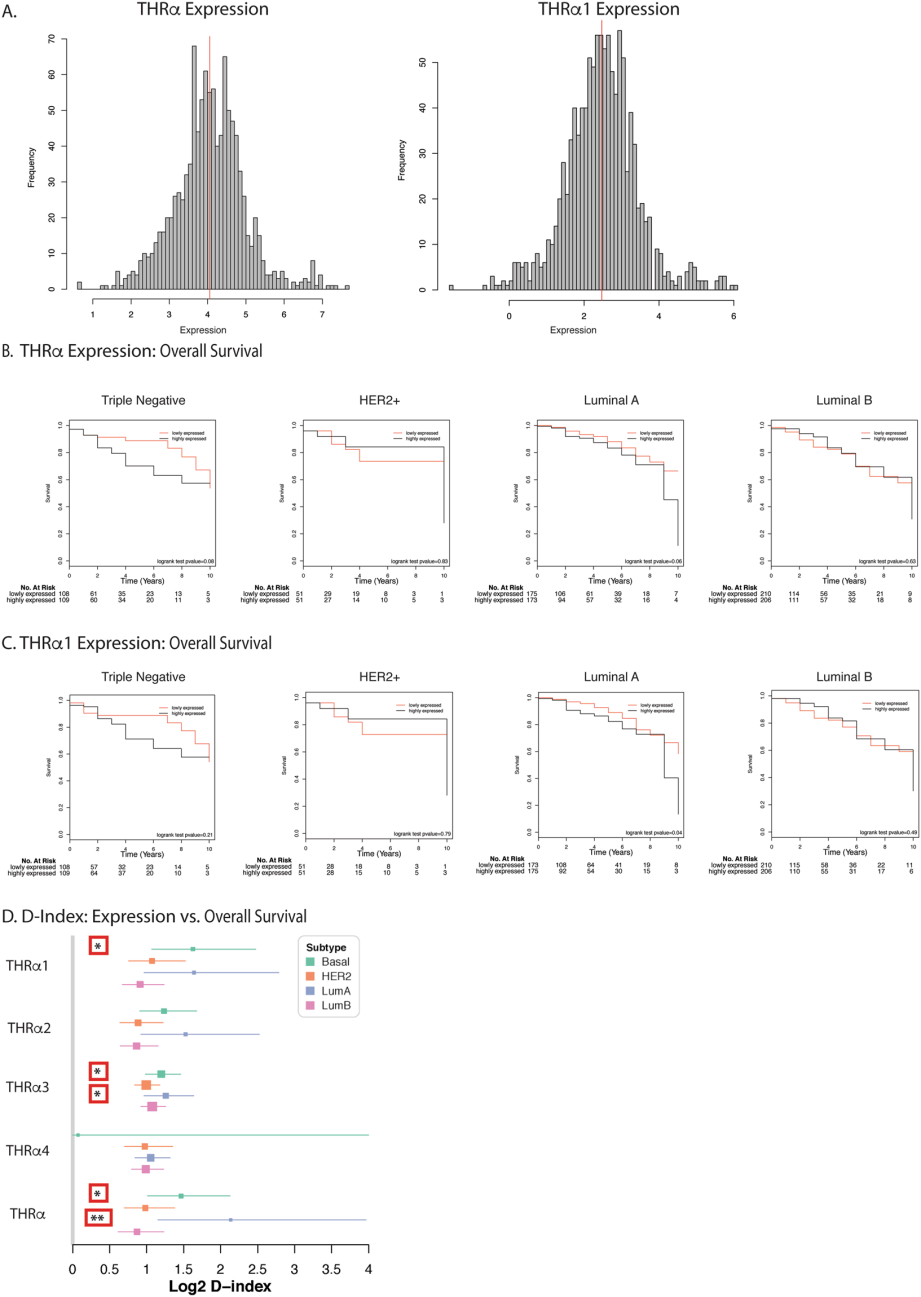
THRα and THRα1 expression is associated with shorter overall survival in breast cancer patients in The Cancer Genome Atlas (TCGA) dataset. (**A**) Histogram depicting the frequency of expression of both THRα and THRα1 in breast cancer patients included in the TCGA dataset (**B**) Kaplan-Meier Survival curve showing proportion of breast cancer patient overall survival in the TCGA dataset with high versus low expression of THRα. No significant difference in overall survival was between patients with low or high expression of THRα (**C**) Kaplan-Meier Survival curve showing proportion of patient overall survival in the TCGA dataset with low versus high expression of THRα1. A statistically significant association is present in the luminal A subtype (p = 0.04). P-values calculated for log-rank test between the two groups (**D**) Forest plot of median with 95% confidence intervals of D-Index of the expression values. D-index indicating prognostic significance treating expression as a continuous variable when evaluating impact on overall survival. Significant values indicated for adjacent confidence intervals; *p < 0.05, **<0.01.

To further assess the clinical relevance of THRα and its isoforms in human breast tumors, The Cancer Genome Atlas (TCGA) data was surveyed (TCGA, TCGA research network). The RNA-seq data of 1099 samples (1092 primary tumors, 7 metastatic) was obtained and further analyzed to characterize the mRNA isoform expression profiles, in addition to gene specific mRNA expression, for each sample. The expression of complete THRα (all isoforms) and THRα1 followed an almost-normal distribution (Fig. 1A). The expression of other isoforms of THRα were also characterized; THRα4 is infrequently expressed in the TCGA breast cancer data (Sup. Fig. 1A). Samples were also classified according to intrinsic subtype, using Genefu^33^. Classically, the luminal A/B subtype is ER+, the HER2 subtype is HER2+, and so-called triple-negative/basal tumors lack the expression of all three receptors. In general, expression of both full length THRα and THRα1, THRα2, and THRα3 were significantly less in the basal (triple negative) subgroup when compared to luminal A/B and HER2 subgroups (p < 0.001, Sup. Fig. 1C,D). HER2 subgroups had significantly higher expression than luminal A/B (p < 0.001, Sup. Fig. 1C,D), while luminal A only had significant elevation in THRα (p = 0.009) and THRα2 when compared to luminal B tumors (p < 0.001, Sup. Fig. 1C,D).

Two approaches were performed to evaluate the prognostic significance of THRα and THRα1. In one analysis, the samples were dichotomized into two groups characterized by low and high expression of the full gene or isoforms. The median expression value was used as the dichotomization cut-off (Fig. 1A). Overall survival was used as the primary outcome and log-rank tests were used for statistical analysis. While trends towards worse overall survival were observed in the triple negative and luminal A subtype cancers expressing high THRα and THRα1 (Fig. 1B,C), this relationship was statistically significant only in the luminal A subtype for THRα1 (Fig. 1C, p = 0.04). No other statistically significant changes in overall survival were observed in patients, classified by intrinsic receptor subtype, with high versus low expression of THRα2, THRα3, or THRα4 (Sup. Fig. 1B).

To address the inherent issues and confounding biases of analysis by dichotomization into low and high expression, analysis was also performed to evaluate the prognostic significance of gene expression as a continuous variable. The D-index was calculated for THRα and all isoforms. This demonstrated that higher expression of both THRα and THRα1 are associated with a decrease in overall survival in women with triple negative (basal like) breast cancer (Fig. 1D), and also identified a novel prognostic association with THRα3 in basal-like and luminal A subtype (Fig. 1D).

### Dronedarone, an FDA-approved drug that antagonizes THRα1, has cytotoxic effects and induces apoptosis in breast cancer cell lines

**Figure 2:**
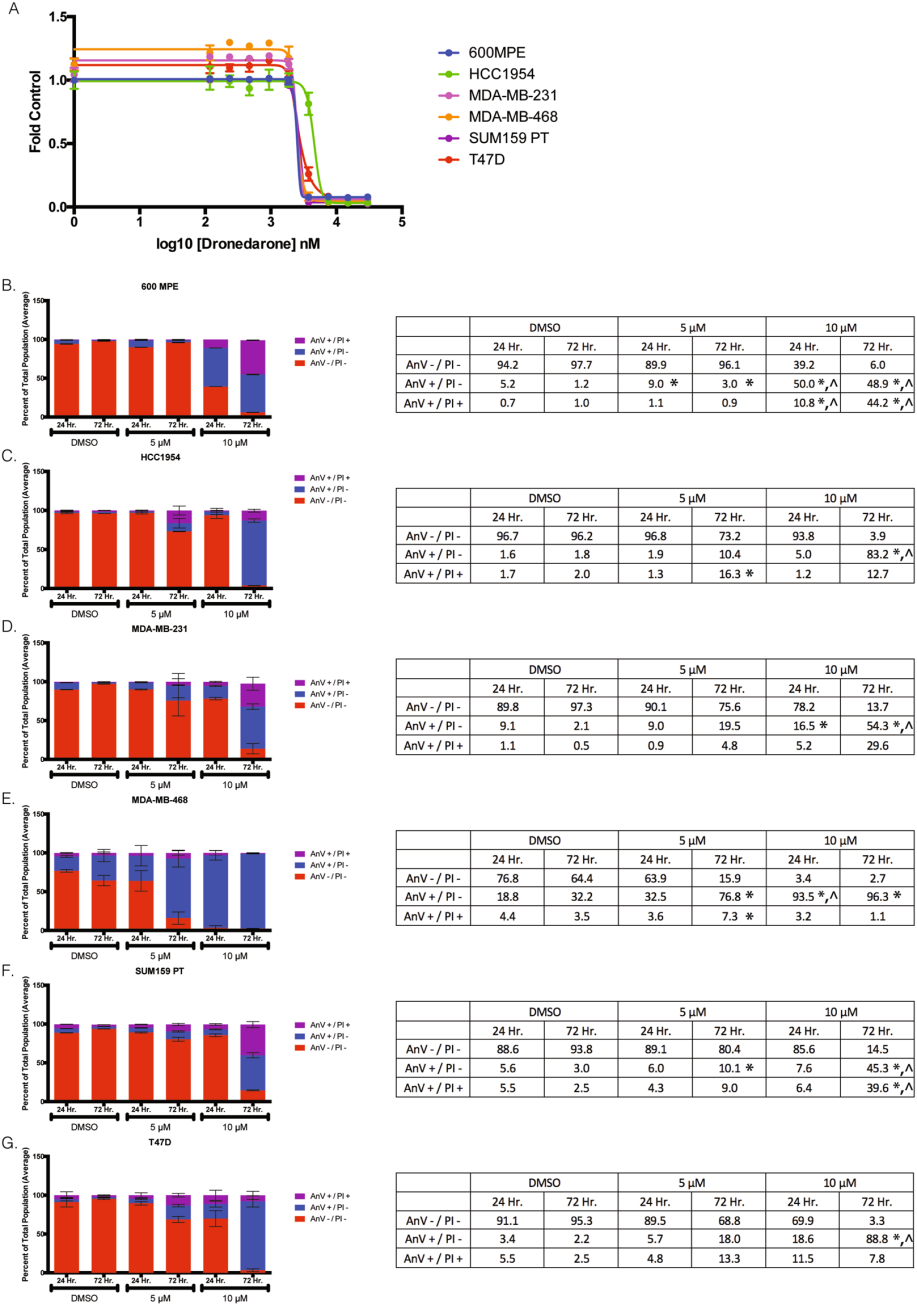
Dronedarone, an FDA-approved drug that antagonizes THRα1 has cytotoxic effects in breast cancer cell lines at relevant concentrations. (**A**) 600 MPE, HCC1954, MDA-MB-231, MDA-MB-468, SUM159 PT, and T47D representative dose response curves. (**B**) 600 MPE (**C**) HCC1954 (**D**) MDA-MB-231 (**E**) MDA-MB-468 (**F**) SUM159 PT and (**G**) T47D at 24 and 72 hours of treatment. Bar graphs represent percentage of total cells unstained or stained with Annexin-V or both Annexin-V and PI. Values representative average percentage of total cell population for each cell population (n = 2). Error bars indicate mean ± standard deviation. Statistical analysis evaluated by two-way ANOVA.

In cardiomyocytes, dronedarone is a known antagonist of THRα1 activity *in vitro* and *in vivo* at clinically relevant concentrations^28^. To determine the effect of dronedarone on breast cancer cells *in vitro*, five-day sulforhodamine B (SRB) dose response assays were performed in eighteen breast cancer cell lines, 600 MPE, AU565, BT20, BT549, CAL120, EVSAT, HCC1395, HCC1937, HCC1954, HS578T, MDA-MB-134, MDA-MB-231, MDA-MB-436, MDA-MB-453, MDA-MB-468, SUM159 PT, SW527, and T47D (Sup. Table 1, Sup. Fig. 2). 600 MPE (luminal B), HCC1954 (Her2+), MDA-MB-231 (triple negative – Basal-like 2 subtype), MDA-MB-468 (triple negative – Basal-like 1 subtype), SUM159 PT (triple negative – mesenchymal stem cell subtype), and T47D (luminal A) were selected to represent the range of intrinsic subtypes (with added emphasis for the triple negative subtype, given the results of the survival analyses and the clinical need in this patient population) for more detailed analysis (Fig. 2A)^34,35^. The observed IC_50_ values for the aforementioned six were 2.91 µM, 4.32 µM, 2.57 µM, 2.33 µM, 2.58 µM, and 2.73 µM respectively; full IC_50_ data and dose response curves are included as supplementary data (Sup. Table 1, Sup. Fig. 2).

The mechanism of dronedarone’s *in vitro* effects was further evaluated in the panel of six representative cell lines. To determine whether this was mediated through the induction of apoptosis, cells were treated with either DMSO or dronedarone at a concentration of 5 µM, or 10 µM for 24 or 72 hours, then collected and subjected to annexin-V/propidium iodide (PI) staining and FACS analysis. Induction of apoptosis was observed in all six cell lines tested, although the degree and timing varied between each cell line. In general, there was a trend towards increases in early and late apoptosis in all cell lines treated with 5 µM and 10 µM of dronedarone at 24 and 72 hours. Amongst the cell lines, the extent and timing of which apoptosis was induced varied. Statistically significant differences between the control (DMSO) and treatment group (5 µM or 10 µM) are indicated (Fig. 2B–G, *p < 0.05). Also, statistically significant differences between the 5 µM and 10 µM at 24 and 72 hours are indicated (Fig. 2B–G, ^p < 0.05).

### Dronedarone has anti-tumor activity in breast cancer xenograft models

**Figure 3:**
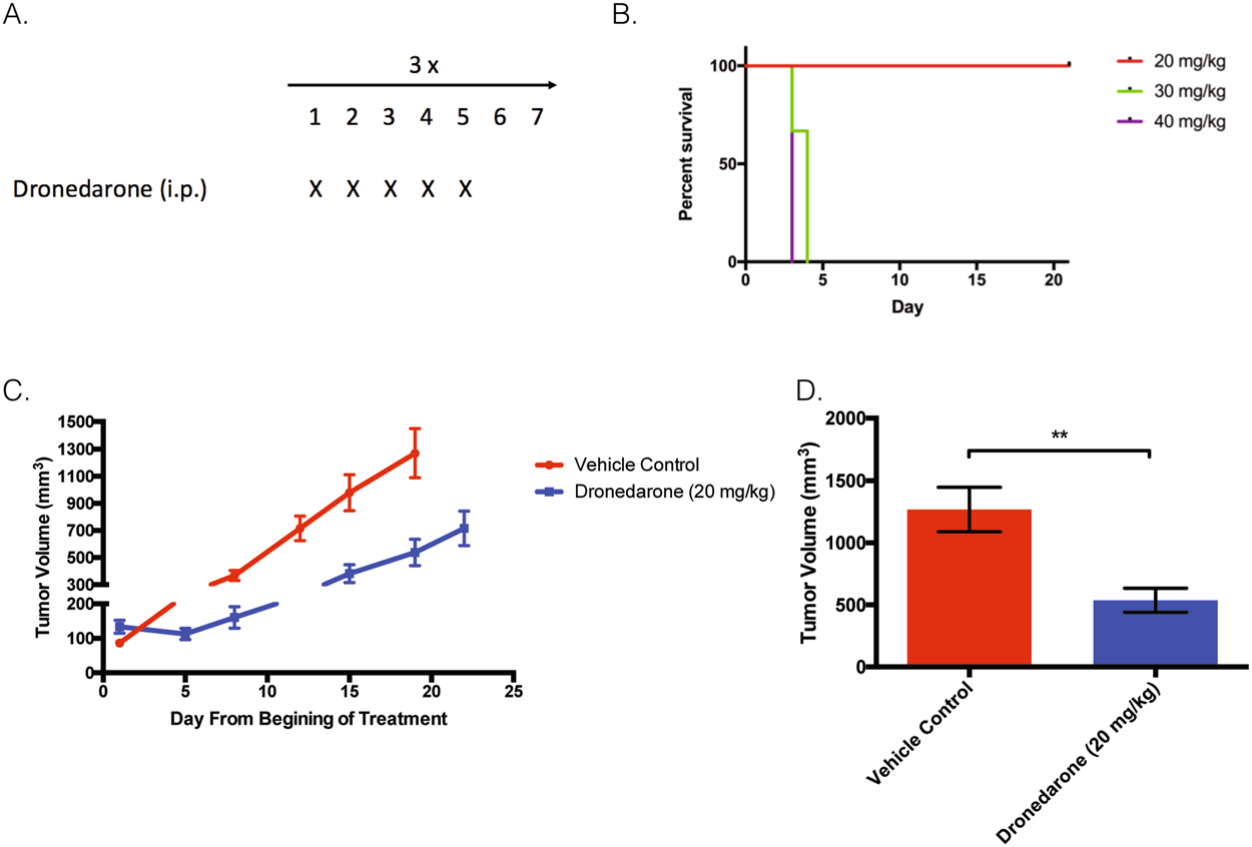
Dronedarone has anti-tumor activity in breast cancer xenograft models. (**A**) Treatment schema for *in vivo* administration of dronedarone (**B**) Kaplan-Meier Survival curve illustrating the overall survival of mice treated with 20 mg/kg, 30 mg/kg, and 40 mg/kg dronedarone (**C**) Tumor volume (mm^3^) measured at indicated time points throughout treatment with dronedarone (20 mg/kg) (**D**) Tumor volume (mm^3^) at day 19 in in groups treated with dronedarone (20 mg/kg). Tumor volume = (π × length × width^2^)/6. Values representative of average of treatment groups (n = 10 per group). P-values indicate significance values for two-tailed Student’s t-tests. All statistics were calculated using GraphPad Prism software. **p < 0.01. Graphs indicate mean ± standard error.

To determine whether dronedarone could inhibit tumor growth *in vivo* in human breast cancer cell lines, at a tolerable and potentially clinically relevant dose, subcutaneous xenografts of the breast cancer cell line HCC1954 were established in NOD/SCID mice. Once tumors reached an average volume of 150 mm^3^, animals were randomized to treatment groups (n = 10) and dronedarone was administered via intraperitoneal injection at 20 mg/kg, 35 mg/kg, or 45 mg/kg for five consecutive days, followed by two days off treatment (Fig. 3A). Treatment was continued for a total of three weeks. The 35 mg/kg and 45 mg/kg doses were not tolerated, with acute toxicity observed (Fig. 3B). However, dronedarone at 20 mg/kg was well tolerated and all mice survived to the predetermined three-week end-point, without significant adverse effects (Fig. 3B). Early sacrifice of animals in the vehicle control group was required at Day 19, because protocol-specified humane endpoints for tumor size were reached. Compared to vehicle, dronedarone treatment resulted in a significant inhibition of tumor growth; average volume in 20 mg/kg treated animals at day 19 was 537.4 mm^3^, compared to 1268.9 mm^3^ in the control group (tumor growth inhibition (TGI) 57.7%; p = 0.01, Fig. 3C,D).

Taxanes are standard of care chemotherapy used for in early and metastatic breast cancer for all disease subtypes. To explore whether dronedarone might have additive activity when combined with taxane chemotherapy, NOD/SCID mice bearing HCC1954 xenografts were treated with dronedarone (20 mg/kg IP, five consecutive days, followed by two days off treatment), docetaxel (10 mg/kg IP once per week) or the combination (n = 10 per group, Sup. Fig. 3A). While the combination was tolerated in most mice (one mouse died mid treatment), when compared to docetaxel (which was effective as a single agent), the addition of dronedarone did not significantly reduce tumor volume at the treatment endpoint (Sup. Fig. 3B). At day 19, tumor volume in the combination group was 60.9 mm^3^, compared to 107.9 mm^3^ in the docetaxel single agent group (Sup. Fig. 3C, p = 0.41). Tumor growth inhibition (TGI) was 96.2% in the combination group as compared to 92.5% in the docetaxel group (Sup. Fig. 3B).

### Depletion of THRα1 or THRα does not affect viability or sensitivity of breast cancer cells to dronedarone-induced cytotoxicity

**Figure 4:**
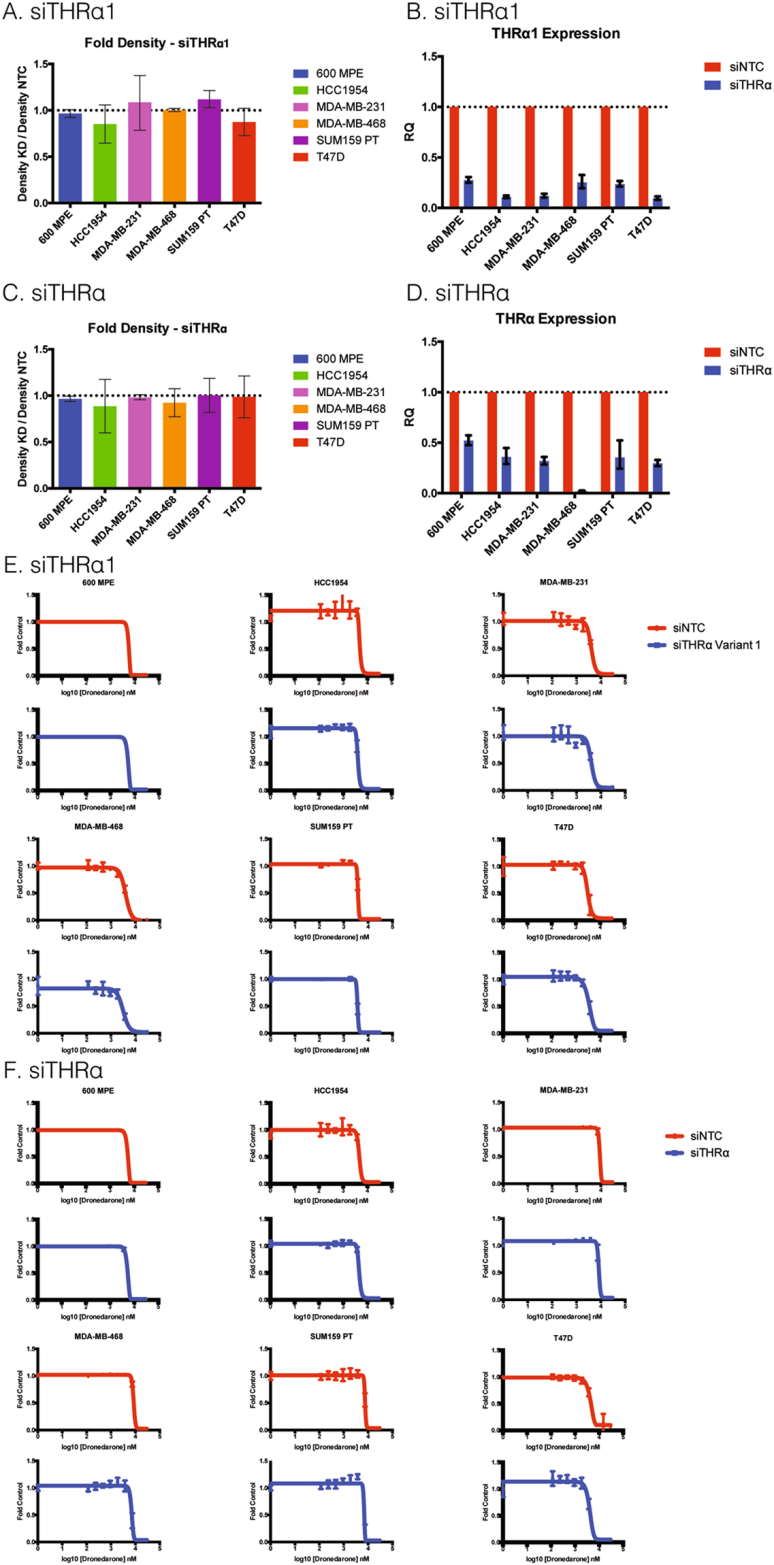
Depletion of THRα1 or THRα does not affect breast cancer cell viability or sensitivity to dronedarone. (**A**) Density of adherent cells assessed with sulforhodamine B (SRB) stain, solubilized, and quantified by spectrophotometry. Relative growth generated by average absorbance of siTHRα1 (knockdown, KD) cells divided by average absorbance of non-targeting control (NTC, n = 12 per group) (**B**) Relative expression of THRα1 measured 48 hours post transfection in 600 MPE, HCC1954, MDA-MB-231, MDA-MB-468, SUM159 PT, and T47D breast cancer cell lines. Measured in relative quantity (RQ) to internal control GAPDH via qRT-PCR. Error bars represent RQ_min_ and RQ_max_. (**C**) Density of adherent cells assessed with sulforhodamine B (SRB) stain, solubilized, and quantified by spectrophotometry. Relative growth generated by average absorbance of siTHRα cells (knockdown, KD) divided by average absorbance of non-targeting control (NTC, n = 12 per group) (**D**) Relative expression of THRα measured 48 hours post transfection in 600 MPE, HCC1954, MDA-MB-231, MDA-MB-468, SUM159 PT, and T47D breast cancer cell lines. Relative quantity (RQ) to internal control GAPDH via qRT-PCR. Error bars represent RQ_min_ and RQ_max_. (**E**,**F**) Representative dose response curves of 600 MPE, HCC1954, MDA-MB-231, MDA-MB-468, SUM159 PT, and T47D breast cancer cells to dronedarone-induced cytotoxicity with or without the knockdown of (**E**) THRα1 or (**F**) THRα. Each value indicates mean (n = 6) ± standard error.

To investigate whether specific depletion of THRα or THRα1 affects breast cancer cell proliferation or viability, as was observed with dronedarone treatment, the six representative cell lines were transfected with siRNA targeting THRα (GE Dharmacon SMARTpool), THRα1 (pool of 4 siRNA, GE Dharmacon), or a non-targeting control. Twenty-four hours following transfection, cells were plated in 96-well plates (denoting day 0) at an optimized initial density of approximately 20% confluency. Cell density was then measured at day five using a sulforhodamine B (SRB) assay. RNA knockdown was confirmed by qRT-PCR analysis 48 hours post transfection in all cell lines tested (Fig. 4B,D). When compared to controls, the relative density of the knockdown compared to the non-targeting control (NTC) was approximately equal to one, with the error bars all crossing the threshold of one. This indicates that there are no meaningful differences in cell density with depletion of either THRα or THRα1 in any of the cell lines tested (Fig. 4A,C).

To assess the specificity of THRα1 knockdown, qRT-PCR was performed using primers specific for each isoform. Interestingly, while knockdown of THRα1 did not significantly alter the mRNA levels of THRα2, 3 or 4 in HCC1954, MDA-MD-231, MDA-MB-468, or T47D cells; the mRNA levels of all variants were reduced in the 600 MPE and SUM159 PT cell line (Sup. Fig. 4A–F).

The fact that knockdown of THRα1 and full length THRα failed to recapitulate the cytotoxic and anti-tumor effects of dronedarone suggests that the anticancer activity of this compound is not simply the consequence of antagonism of THRα1 or THRα. To confirm this, 600 MPE, HCC1954, MDA-MB-231, MDA-MB-468, SUM159 PT, and T47D cells were transfected with either siTHRα1 or siTHRα (all isoforms), then plated in 96-well plates and treated with dronedarone at concentrations ranging from 0.12 µM to 30 µM, as in the earlier experiments, for five days. Successful knockdown of the siTHRα and siTHRα1 target expression was achieved in all cell lines tested (Fig. 4B,D). After five days, SRB assays were performed to generate dose response curves. Knockdown of THRα1 or THRα did not alter the sensitivity to dronedarone for any of the cell lines tested, with similar dose response curves observed for siTHRα1 and siTHRα compared to their respective siRNA controls (Fig. 4E,F), supporting the notion that this target is not responsible for dronedarone’s anti-cancer effects.

## Discussion

Given a growing body of literature which supports the role of THRα and its splice variants as prognostic biomarkers for overall survival among women with breast cancer, we investigated the prognostic significance of THRα and THRα1 expression in the well-annotated TCGA breast cancer cohort. When dichotomized into low and high expression of THRα and THRα1, there was no difference in overall survival in patients with breast cancer, with the exception of high THRα1 expression and decreased overall survival the luminal A breast cancer subtype (p = 0.04). The luminal A subtype-specific prognostic effect of THRα1 supports the data published by^12^ and suggests that T3 acting through THR signaling increases proliferation and enhances estrogen-mediated growth of hormone receptor positive breast cancer cells. As THRα1 is the only THRα splice variant that is hormone sensitive, it supports this finding. Although there are trends that overall survival is shorter in women with high levels of both THRα and THRα1, especially in women with triple negative and luminal A breast cancer, the number of events in each group may limit the power of this analysis. Furthermore, analysis between two binary groups artificially generated from a continuous variable makes the analysis less sensitive for any potential impacts on overall survival. To try and account for this, analysis was also performed treating expression as a continuous value when evaluating prognosis. When the D-index of the expression values, which estimates the log hazard ratio when comparing two equal-sized prognostic groups, was used, both elevations in THRα and THRα1 were associated with decreased overall survival in women with both basal-like (triple negative), and THRα for women with luminal A breast cancer. Other novel associations with THRα3 in basal-like (triple negative) and luminal A, in addition to THRα in luminal A breast cancer were also identified.

Given this data and previously published data, we sought to experimentally test the hypothesis that inhibition of THRα1 has cytotoxic effects in breast cancer cells. The availability of a clinically-approved drug, dronedarone, that has been shown to antagonize THRα1 presented a promising opportunity to explore the potential of drug repurposing. Our initial experiments using this agent, both *in vitro* and *in vivo*, supported this concept, whereby dronedarone induced apoptosis and was cytotoxic in a dose-dependent manner in eighteen breast cancer cell lines. In addition, these results were further substantiated by the demonstration of meaningful tumor growth inhibition *in vivo*. It is notable that this effect was achieved in HCC1954, a cell line that displayed one of the highest *in vitro* IC50 values. Furthermore, that the dose used (20 mg/kg) was well-tolerated and falls well within the range of doses characterized in non-clinical studies to support dronedarone’s antiarrhythmic indication^36^. Thus, this dose may be clinically relevant in humans.

In an attempt to validate whether the cytotoxic effects of dronedarone are mediated through THRα1, or even THRα, siRNA to knockdown of both targets was pursued in a variety of breast cancer cell lines representing the different intrinsic receptor subtype and molecular backgrounds, including well-characterized luminal A, luminal B, Her2+, and triple negative (basal-like) breast cancer cell lines, 600 MPE, HCC1954, MDA-MB-231, MDA-MB-468, SUM159 PT, and T47D. These experiments were technically successful yet did not demonstrate that THRα1 or THRα mRNA depletion impaired breast cancer cell growth or survival. The absence of an effect on cell survival with THRα or THRα1 knockdown is consistent with large scale functional genomic studies that demonstrate the gene is not essential in breast or other cancer cells^37^.

Using the same well-characterized cell lines, we assessed whether modulation of THRα1 or THRα had any impact on dronedarone activity by combining gene knockdown with the putative pharmacologic inhibitor. These experiments showed that the cytotoxic effect of dronedarone was independent of and not altered by THRα or THRα1 knockdown in all cell lines tested; this finding provides evidence to support that THRα or THRα1 is not the target that mediates dronedarone’s anti-cancer effects. Taken together, our results demonstrate that although dronedarone is cytotoxic both *in vitro* and *in vivo*, this effect is not dependent on THRα or THRα1.

Dronedarone has been shown to have multiple pharmacologic effects, including inhibition of beta-adrenergic receptors and multiple transmembrane potassium currents in addition to the inward depolarizing sodium and L-type calcium currents^38^. Calcium influx, in particular, is well known to regulate several intracellular pathways in cancer cells^39^. Recently, dronedarone has also been shown to induce DNA-damage and apoptosis in a hepatocyte model through the downregulation of topoisomerase IIα at the transcriptional and post-transcriptional level leading to the activation of caspase-2 and downstream JNK and p38 signalling pathways^40^. Thus, while one or more of multiple discrete mechanisms may contribute to dronedarone’s anticancer effects, our *in vitro* and *in vivo* data would support the further investigation of this compound or related derivatives in order to characterize the basis of these effects. The identification of the principal pharmacologic targets mediating its activity could create new opportunities for drug repurposing or the development of more selective novel therapies.

## Materials and Methods

### Clinical Information and gene expression analyses

TCGA transcript level quantifications and clinical data for 1092 breast cancer patients were downloaded from *UCSC Xena* browser (https://xenabrowser.net/). RNA-seq raw data is quantified with *Kallisto*^41^ in *Toil* pipeline^42^ using the GENCODE version 23 (ALL version) transcriptome annotation. Transcript level abundances are summarized to gene-level using the same approach as described in^43^.

### Cell culture

Breast cancer cell lines from the American Type Culture Collection (ATCC) were maintained in specific media according to ATCC recommendations. Cells were incubated at 37 °C with 5% CO2 supplementation and passaged routinely when required.

### Dronedarone Dose Response Curves

Cells were seeded in 96 well plates at optimized cell densities (approximately 20% confluency) and treated with dronedarone maintained in a stock solution dissolved in dimethyl sulfoxide (DMSO) at 10 mM. Working concentrations were made using 2-fold dilutions, diluted in cell culture media. After 5 days, cell viability was assessed via sulforhodamine B (Sigma) assay.

### Apoptosis analysis

Cells were plated in 6 well plates and treated 24 h later with dronedarone or vehicle control. At specified time points cells were washed, collected, and stained with Annexin-V (AnV)-FITC (BioLegend) and propidium iodide (PI, Sigma) and measured on a BD FACS Canto II flow cytometer. Spectral compensation was performed. FloJo 10.1 software was used to quantify the proportion of AnV+/PI− and AnV+/PI+ cells.

### RNA isolation and cDNA Synthesis

Cells were pelleted, and RNA was extracted using the NucleoSpin® RNA Purification Kit (Macherey-Nagel) as per manufacturer instructions. cDNA was generated using reverse transcription reactions (iScript, Bio-Rad) from 2 μg of total RNA.

### RT-qPCR

Custom qRT-PCR primers were designed for the THRα splice variants using the NCBI Primer Design Software (NIH). THRα splice variant 2 and 4 share significant homology and the software generated a single forward and reverse primer for the isoforms. Real-time qPCR was performed using SYBR Green Mastermix (Applied Biosystems) on an Applied Biosystems 7900 HT Real-Time PCR machine with a 384-well block in triplicate. Data were collected and analyzed using the ΔΔ*C*_t_ method with GAPDH as the reference gene.

**Table:** 

Target		
THRα Splice Variant 1	Forward	TCCGACGCCATCTTTGAACT
	Reverse	TCATGCGGAGGTCAGTCAC
THRα Splice Variant 2 and 4	Forward	ACCGCAAACACAACATTCCG
	Reverse	ATTCCGAGAAGCTGCTGTCC
THRα Splice Variant 3	Forward	CCAAGCTGCTGATGAAGGTG
	Reverse	CTTGGAGACTTCCCGCTTCAC
GAPDH	Forward	GGAAGCTCACTGGCATGGCC
	Reverse	CCTGCTTCACCACCTTCTTG

### RNA Interference

siRNA specific to THRα splice variant 1 (GE Dharmacon) and THRα full length (Amersham) were purchased. Appropriate volumes of siRNA were suspended in Lipofectamine 3000 (Invitrogen, Life Technologies) and Opti-MEM (Thermofisher) at a final concentration of 100 nM and 50 nM for siTHRα1 and siTHRα respectively. 24 hours after transfection, cells were trypsinized, counted, and plated. Gene and splice-variant specific knockdown was determined using qRT-PCR.

**Table:** 

THRα1 (NM_199334)	GGAGAAGACAAAUGAAGAA
	GGGAGAAGACAAAUGAAGA
	GAGAAGACAAAUGAAGAAA
	GGAGGAUUGAGAAGGGACA

### *In*-*vivo* studies

HCC1954 cell line xenografts were established in NOD/SCID mice by subcutaneous injection of 50,000 cells. Mice were randomized to treatment with docetaxel (10 mg/kg), dronedarone (20 mg/kg, 35 mg/kg, and 45 mg/kg), and combination vs. vehicle via an intraperitoneal (IP) route when tumors reached a mean volume of approximately 150 mm (n = 10/group). Docetaxel was administered once per week, and dronedarone was administered on five consecutive days, followed by two days off treatment. Dronedarone and docetaxel were co-administered on day one, eight, and fifteen in the combination group. Vehicle control consisted of 5% EtOH, 12.5% DMSO, 12.5% Tween20, 70% PBS. Tumor growth was monitored by caliper measurements, during 3 weeks of treatment, following which tumors were harvested and weighed at the end of the experiment. All animal studies were performed in accordance with the animal care guidelines published by McMaster University Animal Research Ethics Board.

### Data Analysis and Statistics

Data and statistical methods are expressed as outlined in figure legends. Standard statistical methods were performed using Prism 6 GraphPad® software.

### Ethical Approval

All animal studies were approved by the McMaster University Animal Research Ethics Board prior to conducting the experiments. All animal studies were performed in accordance with the animal care guidelines published and enforced by the McMaster University Animal Research Ethics Board.

## Electronic supplementary material

Supplementary Table and Figures

## Data Availability

The datasets generated during and/or analyzed during the current study are available from the corresponding author on reasonable request.
