# Supplementary material for: The Antiarrhythmic Drug, Dronedarone, Demonstrates Cytotoxic Effects in Breast Cancer Independent of Thyroid Hormone Receptor Alpha 1 (THRα1) Antagonism
Source: Sci Rep. 2018 Nov 8;8:16562. doi: 10.1038/s41598-018-34348-0 (PMC6224430; doi:10.1038/s41598-018-34348-0)
Supplement: Supplementary file 1 — Supplementary Table and Figures [file 41598_2018_34348_MOESM1_ESM.pdf]

The Antiarrhythmic Drug, Dronedarone, Demonstrates Cytotoxic Effects in Breast Cancer Independent of Thyroid Hormone Receptor Alpha 1 (THRα1) Antagonism

Authors:

Mitchell J. Elliott[⊥1], Katarzyna Jerzak[⊥2,3], Jessica G. Cockburn[4], Zhaleh Safikhani[1], William Gwynne[4], John Hassell[4], Anita Bane[4], Jennifer Silvester[1], Kelsie Thu[1], Benjamin Haibe-Kains[1], Tak W. Mak[1], David Cescon*[1,3]

*Corresponding author (dave.cescon@uhn.ca)
[⊥]Co-first authors

[1] The Princess Margaret Cancer Centre, University Health Network, Toronto, Canada
[2] Sunnybrook Health Science Centre, Odette Cancer Centre, Toronto, Canada
[3] Division of Medical Oncology, Department of Medicine, University of Toronto, Toronto, Canada
[4] McMaster University, Hamilton, Canada

Supplementary Table 1:

Cell Line	IC_{50} Value (μM)
600MPE	2.91
AU565	4.00
BT20	2.14
BT549	2.91
CAL120	3.44
EVSA-T	2.72
HCC1395	3.16
HCC1937	2.90
HCC1954	4.32
HS578T	3.45
MDA-MB-134	5.61
MDA-MB-231	2.57
MDA-MB-436	3.65
MDA-MB-453	2.25
MDA-MB-468	2.33
SUM159 PT	2.58
SW527	2.34
T47D	2.73

Supplementary Table 1: Dronedarone, an FDA-approved drug that antagonizes THRα1 has cytotoxic effects in breast cancer cell lines. (A) Average IC_{50} values (nM) listed as average by cell line for independent biological replicates (n=2). Cells were treated with concentrations of dronedarone ranging from 0.12 μM to 30 μM for five days. Density of adherent cells after 5 days of treatment was assessed with sulforhodamine B (SRB) stain, solubilized, and quantified by spectrophotometry. Relative growth generated by average absorbance of treated divided by average absorbance of DMSO treated control.

A.

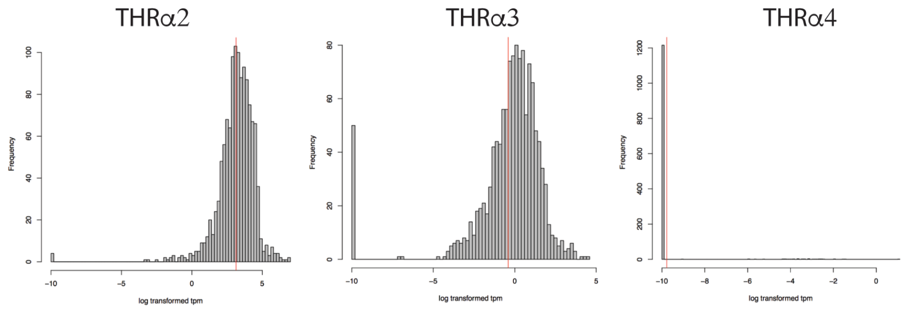

B.

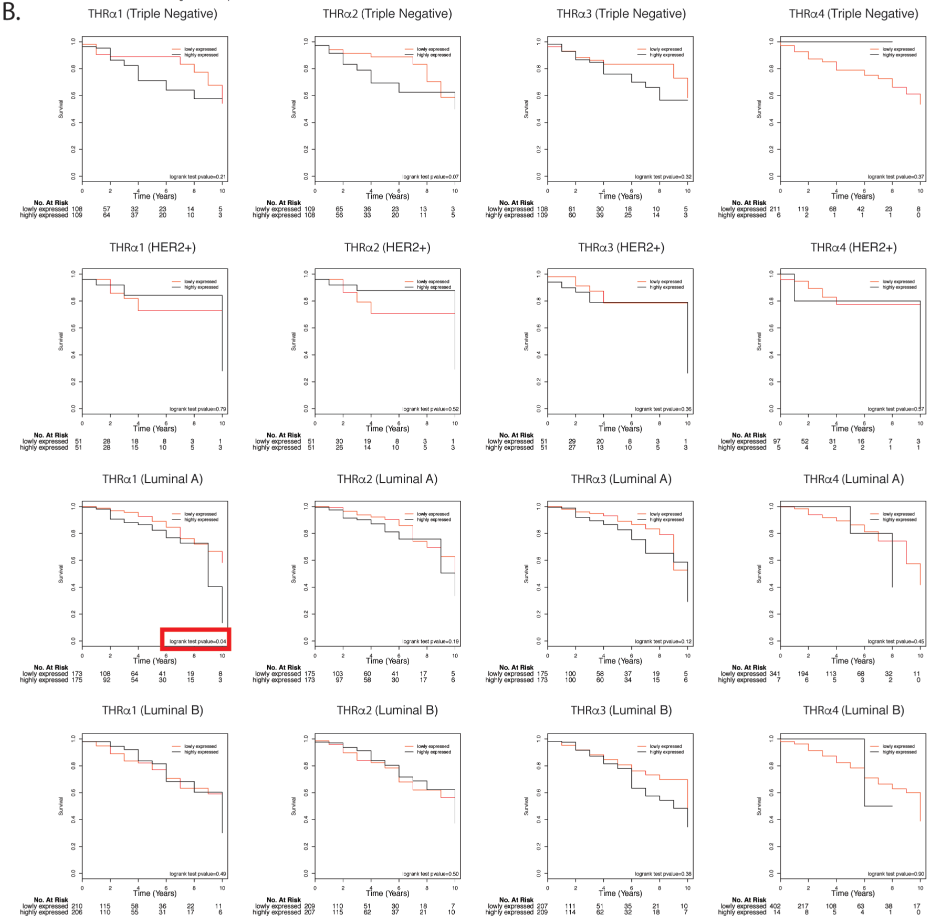

C.

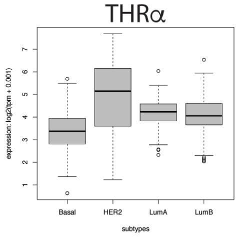

	p-value
Basal < HER2	1.43E-16
Basal < LumA	4.81E-33
Basal < LumB	3.45E-23
HER2 > LumA	2.23E-07
HER2 > LumB	9.81E-09
LumA > LumB	0.00931855

D.

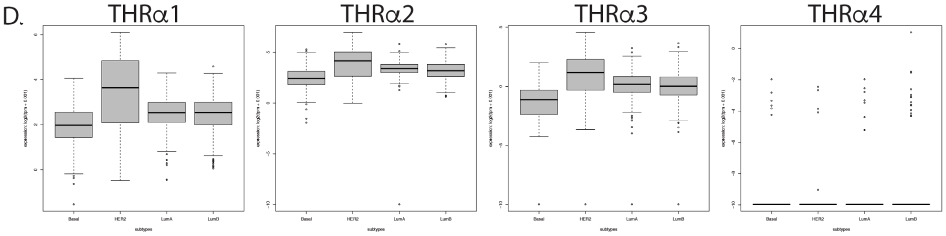

	p-value
Basal < HER2	4.55E-15
Basal < LumA	5.52E-15
Basal < LumB	2.64E-13
HER2 > LumA	8.91E-09
HER2 > LumB	2.50E-09
LumA < LumB	0.64164464

	p-value
Basal < HER2	1.85E-15
Basal < LumA	1.35E-32
Basal < LumB	2.76E-20
HER2 > LumA	2.55E-05
HER2 > LumB	3.19E-07
LumA > LumB	9.78E-05

	p-value
Basal < HER2	4.60E-20
Basal < LumA	1.29E-30
Basal < LumB	1.25E-24
HER2 > LumA	8.53E-08
HER2 > LumB	3.75E-09
LumA > LumB	0.11261881

	p-value
Basal < HER2	0.33836723
Basal < LumA	0.57360559
Basal < LumB	0.67239762
HER2 < LumA	0.11714604
HER2 < LumB	0.4765164
LumA < LumB	0.25583991

Supplementary Figure 1. THRα and THRα1 overexpression is associated with shorter overall survival in breast cancer patients in The Cancer Genome Atlas (TCGA) dataset. (A) Histograms depicting expression of THRα2, THRα3, and THRα4 in breast cancer samples in the TCGA dataset. Samples adjusted for log transformed TPM (transcript per kilobase million) (B) Kaplan-Meier Survival curve showing proportion of breast cancer patient overall survival in the TCGA dataset with high versus low expression of THRα1, THRα2, THRα3, and THRα4 based on three-gene classifier subtype (Basal/TNBC, HER2+, Luminal A/B). P-values calculated for log-rank test between the two groups (C) Box plot representing average expression of THRα in breast cancer patients in the TCGA dataset separated by intrinsic receptor subtype. Graph shows lower and upper extremes, lower and upper quartile, and median. P-values amongst caparisons illustrated in adjacent chart. (D) Box plot representing average expression of THRα1, THRα2, THRα3, and THRα4 in breast cancer patients in the TCGA dataset separated by intrinsic receptor subtype. Graph shows lower and upper extremes, lower and upper quartile, and median. P-values amongst comparisons illustrated in accompanying chart.

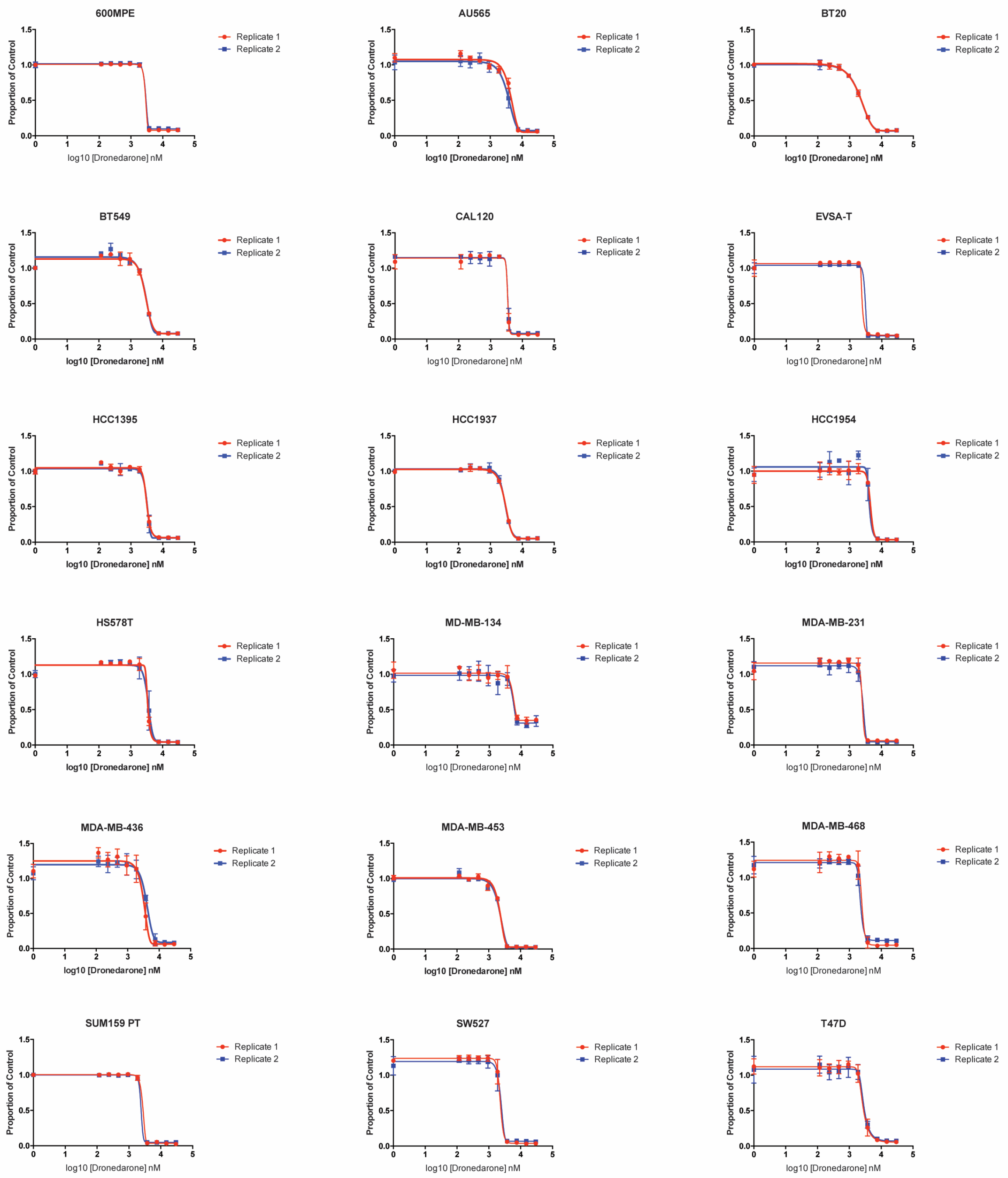
600MPE
AU565
BT20
BT549
CAL120
EVSA-T
HCC1395
HCC1937
HCC1954
HS578T
MD-MB-134
MDA-MB-231
MDA-MB-436
MDA-MB-453
MDA-MB-468
SUM159 PT
SW527
T47D
Replicate 1
Replicate 2
Proportion of Control
log10 [Dronedarone] nM

Supplementary Figure 2. Dronedarone, an FDA-approved drug that antagonizes THRα1 has cytotoxic effects in breast cancer cell lines at relevant concentrations. Representative dose response curves for eighteen breast cancer cell lines treated with 0.12 μM to 30 μM of dronedarone for five days. Density of adherent cells after 5 days of treatment was assessed with sulforhodamine B (SRB) stain, solubilized, and quantified by spectrophotometry. Average IC_{50} of biological replicates (n=2) are found in supplementary table 1.

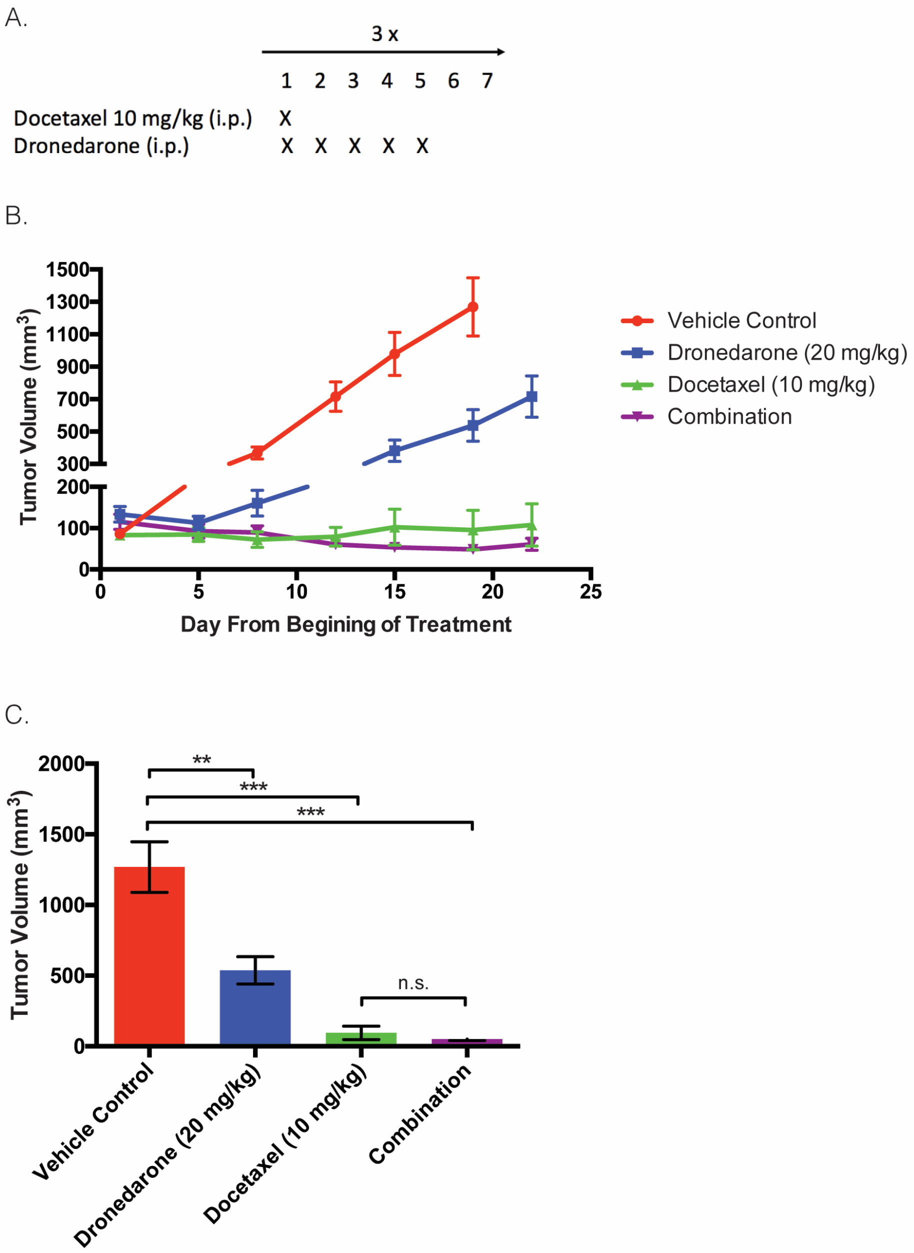
A.
3 x
1 2 3 4 5 6 7
Docetaxel 10 mg/kg (i.p.) X
Dronedarone (i.p.) X X X X X
B.
Tumor Volume (mm^3)
1500
1300
1100
900
700
500
300
200
100
0
0 5 10 15 20 25
Day From Begining of Treatment
Vehicle Control
Dronedarone (20 mg/kg)
Docetaxel (10 mg/kg)
Combination
C.
Tumor Volume (mm^3)
2000
1500
1000
500
0
**
***
***
n.s.
Vehicle Control
Dronedarone (20 mg/kg)
Docetaxel (10 mg/kg)
Combination

Supplementary Figure 3. Dronedarone has anti-tumor activity in breast cancer xenograft models. (A) Schematic representing dosing schedule for the intraperitoneal administration of dronedarone (20 mg/kg), docetaxel (10 mg/kg) or in combination, for three weeks. (B) Tumor volume (mm^3) measurement at indicated timepoints throughout treatment protocol. NOD/SCID mouse xenografts of HCC1954 breast cancer cells line treated with vehicle control, dronedarone (20 mg/kg; n=10), docetaxel (10 mg/kg; n=10), or in combination (n=9). (C) Comparison of tumor volumes (at day 19). P-values indicate significance values for two-tailed Student's t-tests. All statistics were calculated using GraphPad Prism software. * $p<0.05$, ** $p<0.01$, *** $p<0.001$, **** $p<0.0001$. Graphs indicate mean ± standard error.

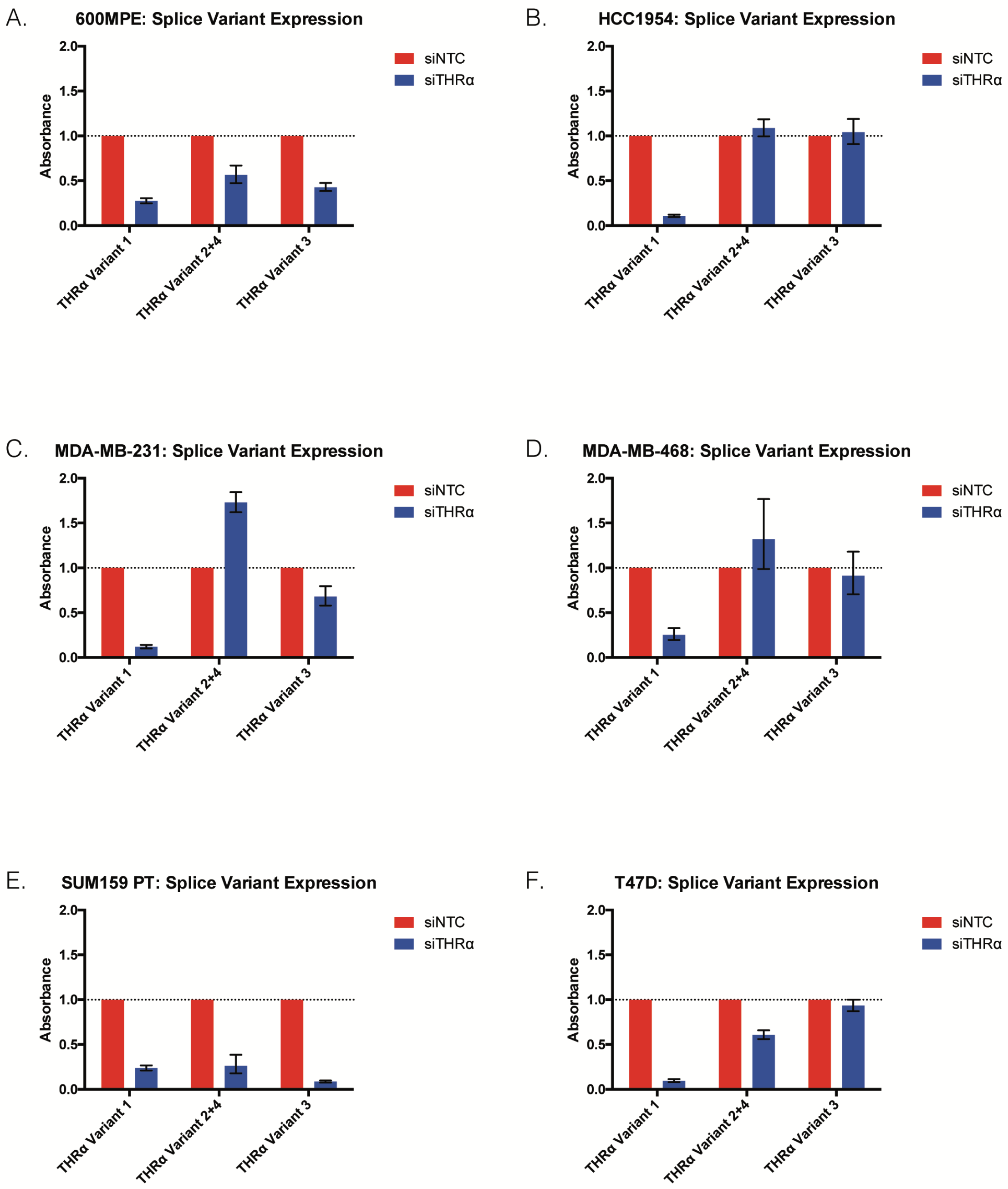
A.
600MPE: Splice Variant Expression
B.
HCC1954: Splice Variant Expression
C.
MDA-MB-231: Splice Variant Expression
D.
MDA-MB-468: Splice Variant Expression
E.
SUM159 PT: Splice Variant Expression
F.
T47D: Splice Variant Expression
Absorbance
0.0
0.5
1.0
1.5
2.0
THRα Variant 1
THRα Variant 2+4
THRα Variant 3
siNTC
siTHRα

Supplementary Figure 4. Depletion of THRα1 or THRα does not affect breast cancer on cell viability or sensitivity to dronedarone induced cytotoxicity. (A-F) Representative illustration of THRα splice variant expression. Relative quantity (RQ) is listed as relative to internal control GAPDH. Splice variants were measured using qRT-PCR analysis after knockdown of target gene splice variant.
